# EEG Reactivity Predicts Individual Efficacy of Vagal Nerve Stimulation in Intractable Epileptics

**DOI:** 10.3389/fneur.2019.00392

**Published:** 2019-05-02

**Authors:** Milan Brázdil, Irena Doležalová, Eva Koritáková, Jan Chládek, Robert Roman, Martin Pail, Pavel Jurák, Daniel J. Shaw, Jan Chrastina

**Affiliations:** ^1^Departments of Neurology and Neurosurgery, Medical Faculty of Masaryk University, Brno Epilepsy Center, St. Anne's University Hospital, Brno, Czechia; ^2^Behavioral and Social Neuroscience Research Group, CEITEC–Central European Institute of Technology, Masaryk University, Brno, Czechia; ^3^Institute of Biostatistics and Analyses, Faculty of Medicine, Masaryk University, Brno, Czechia; ^4^Institute of Scientific Instruments, The Czech Academy of Sciences, Brno, Czechia

**Keywords:** vagal nerve stimulation, neurostimulation, epilepsy, efficacy prediction, EEG reactivity, epilepsy treatment

## Abstract

**Background:** Chronic vagal nerve stimulation (VNS) is a well-established non-pharmacological treatment option for drug-resistant epilepsy. This study sought to develop a statistical model for prediction of VNS efficacy. We hypothesized that reactivity of the electroencephalogram (EEG) to external stimuli measured during routine preoperative evaluation differs between VNS responders and non-responders.

**Materials and Methods:** Power spectral analyses were computed retrospectively on pre-operative EEG recordings from 60 epileptic patients with VNS. Thirty five responders and 25 non-responders were compared on the relative power values in four standard frequency bands and eight conditions of clinical assessment—eyes opening/closing, photic stimulation, and hyperventilation. Using logistic regression, groups of electrodes within anatomical areas identified as maximally discriminative by *n* leave-one-out iterations were used to classify patients. The reliability of the predictive model was verified with an independent data-set from 22 additional patients.

**Results:** Power spectral analyses revealed significant differences in EEG reactivity between responders and non-responders; specifically, the dynamics of alpha and gamma activity strongly reflected VNS efficacy. Using individual EEG reactivity to develop and validate a predictive model, we discriminated between responders and non-responders with 86% accuracy, 83% sensitivity, and 90% specificity.

**Conclusion:** We present a new statistical model with which EEG reactivity to external stimuli during routine presurgical evaluation can be seen as a promising avenue for the identification of patients with favorable VNS outcome. This novel method for the prediction of VNS efficacy might represent a breakthrough in the management of drug-resistant epilepsy, with wide-reaching medical and economic implications.

## Introduction

Resective surgery is currently the best therapeutic option for treatment of patients with drug-resistant epilepsy, but a substantial number of intractable patients remains who are ineligible for such treatment or for whom resective surgery fails to abolish seizures. Chronic vagal nerve stimulation (VNS) has become a well-established alternative, offering a palliative method of treatment for drug-resistant epilepsy; it rarely results in complete seizure freedom (~5% of treated patients), but provides substantial (≥50%) seizure reduction in 50–60% of individuals. Unfortunately, however, seizure frequency remains unchanged after VNS therapy in ~25% of patients (1, 2).

Identifying individuals who will benefit from VNS therapy prior to the implantation of the VNS device would improve patient selection, minimize unnecessary surgical procedures, and reduce associated financial expenses dramatically; and yet there exists no method with which to predict individual efficacy pre-intervention (2). Achieving a pre-operative classification of individual patients as VNS responders or non-responders (i.e., patients with ≥50% or <50% seizure reduction, respectively) would represent a major breakthrough in the treatment of drug-resistant epilepsy.

It is presumed that VNS increases seizure threshold by activating neuronal networks in the thalamus and other limbic structures (3, 4), but the precise mechanism of VNS action is not yet understood fully. Both synchronization and desynchronization of the electroencephalogram (EEG) has been proposed as a possible mechanism behind the antiepileptic effect of VNS (5, 6), and recent neurophysiological studies focusing on EEG parameters lend support to this: Fraschini et al. report a significant correlation between VNS-induced global desynchronization in gamma bands and positive clinical outcome in temporal lobe epilepsy patients (7). Similarly, Bodin et al. revealed a lower level of global EEG synchronization in delta and alpha frequency bands during the ON phase of VNS in responders (8). Theoretically, differential alterations in brain rhythms from VNS therapy between responders and non-responders might reflect inter-individual variability in the (non-specific) susceptibility of EEG to be synchronized or desynchronized by external stimulation. It follows that differences in this susceptibility might underlie individual VNS efficacy.

We tested the hypothesis that EEG reactivity to standard external stimuli used during routine pre-operative EEG assessment differs between VNS responders and non-responders, with the aim of developing a reliable statistical model for prediction of VNS efficacy.

## Materials and Methods

### Study Design

We performed retrospective analyses of EEG data collected from all adult patients implanted with a VNS device for drug-resistant epilepsy in the Brno Epilepsy Center between 2005 and 2015. Data from patients implanted between 2005 and 2012 were used for investigation of EEG reactivity to external stimuli and subsequently for development of the statistical model **(Cohort 1)**. Additional data from patients implanted with VNS between 2013 and 2015 were used as independent data-set for validation of the statistical model **(Cohort 2)**. All the data were acquired during routine outpatient pre-operative assessment, 20 min recording at morning with two standard eyes opening and closing activation procedures (i.e., 10 s period with eyes open), photic stimulation (PS), and hyperventilation (HV). Each EEG recording was filtered into individual frequency bands and segmented into specific intervals representing the eyes opening and closing, PS and HV periods. The relative powers of EEG spectrum in distinct time intervals for a particular frequency band and brain area were then calculated.

Based on their individual responses to VNS, patients were categorized as Responders or Non-responders. In Cohort 1, Responders and Non-responders were first compared on the relative power values, and then a statistical model for prediction of VNS efficacy was developed. Subsequently, the validity of the statistical model was tested in Cohort 2. The study was conducted in St. Anne's University Hospital and approved by the local ethics committee. All patients gave their informed written consent for the use of their pre-operative data.

### Patients' Description

All patients were implanted with a VNS system (Cyberonics, Houston) according to a standard implantation procedure (9). Before implantation, all patients underwent a comprehensive assessment protocol for epilepsy surgery candidates, including a detailed history and neurological examination, magnetic resonance imaging (MRI), interictal PET, neuropsychological testing, and scalp video-EEG monitoring. In some patients, ictal and interictal SPECT (SISCOM) and invasive video-EEG monitoring have been completed if necessary. Based on the results of all the investigations, patients indicated for VNS and included in this study were ruled out as suitable candidates for resective epilepsy surgery. Our analyses were applied to data acquired from patients who fulfilled the following criteria: The duration of VNS treatment was at least 2 years; the efficacy of VNS treatment was determined in regular visits every 3 or 6 months; and artifact-free pre-operative interictal EEG was available for eye opening and closing, PS and HV periods.

Demographic information and data regarding the type and number of antiepileptic drugs (AEDs) at the time of implantation were obtained by review of patients' charts. The efficacy of VNS was categorized using a classification system reported by McHugh (10). The cut-off value for seizure-reduction between responders and non-responders was 50%. Patients were defined as Responders or Non-responder only if they were categorized as such for the entire follow-up period.

### EEG Analysis

**Figure 1:**
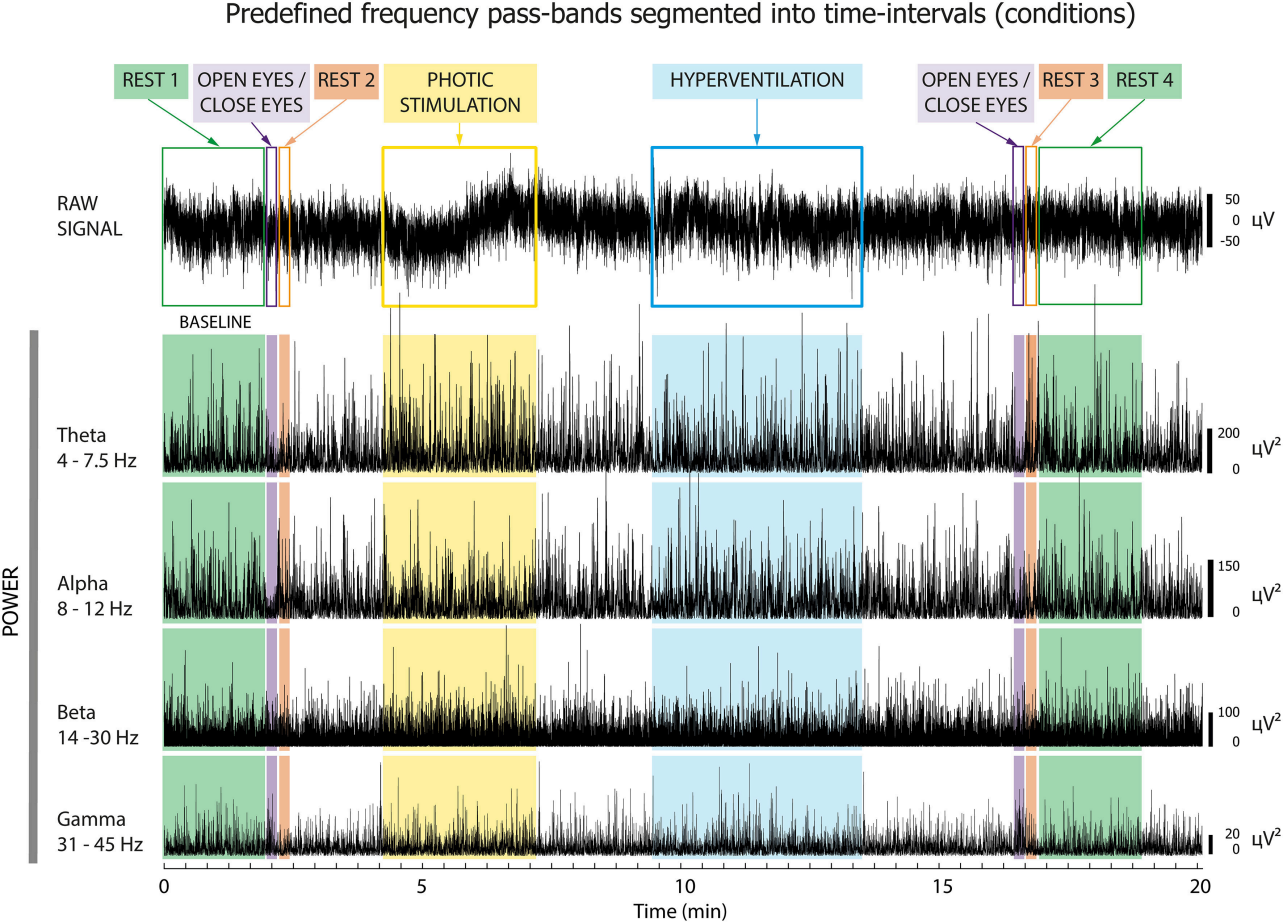
Pre-processing of EEG signal. The EEG was segmented into eight time intervals, and then filtered into four frequency bands: theta, alpha, beta, and gamma. Subsequent analyses focused on the oscillatory power changes within these intervals, which were analyzed separately for each frequency band.

First, we compared relative EEG powers between Responders and Non-responders in Cohort 1. Interictal scalp EEG was recorded on a 64-channel Alien Deymed system with international 10–20 electrode placement and a sampling frequency of 128 Hz. Standard antialiasing filters were applied before digitalization. Occasional artifacts were rejected manually and further processing was performed with artifact-free EEG periods. The resulting EEG signals were filtered into four frequency bands: theta (4–7.5 Hz), alpha (8–12 Hz), beta (14–30 Hz), and gamma (31–45 Hz). A Hilbert transform was then used to estimate the envelopes of pre-defined pass-band frequency oscillations as a function of time (Figure 1). The EEG records were segmented into the following conditions (time intervals):

–Rest#1 (2 min)
–Eyes opening/closing (10 s)
–Rest#2 (immediately after eye closure; 10 s)
–Photic stimulation (PS; 2.5 min)
–Hyperventilation (HV; 4 min)
–Eyes opening/closing (10 s)
–Rest#3 (immediately after eye closure; 10 s)
–Rest#4 (2 min)

Further analysis was focused on oscillatory power changes in these conditions. Absolute mean power of the EEG spectrum was computed as a mean value of the passband power envelope inside each interval separately, for each scalp electrode. Subsequently, relative mean power (RPW) was calculated as a percentage decrease or increase of mean power relative to baseline, i.e., event-related desynchronization or synchronization, respectively. As a baseline we selected Rest#1 (11). We then evaluated differences between Responders and Non-responders by comparing relative power in seven conditions of clinical assessment: Open/Close#1, Rest#2, PS, HV, Open/Close#2, Rest#3, and Rest#4.

### Statistical Analysis

Demographic data were compared between Responders and Non-responders using Fisher's exact test or the Mann-Whitney test. Statistical comparisons of RPW between Responders and Non-responders were also performed with Mann-Whitney tests. Using false discovery rate (FDR) (12), *p*-values for all electrodes were corrected for multiple comparisons in each time interval separately. Differences were considered significant when *p* ≤ 0.05.

### Prediction of Response to VNS

#### Developing the Statistical Model

**Figure 2:**
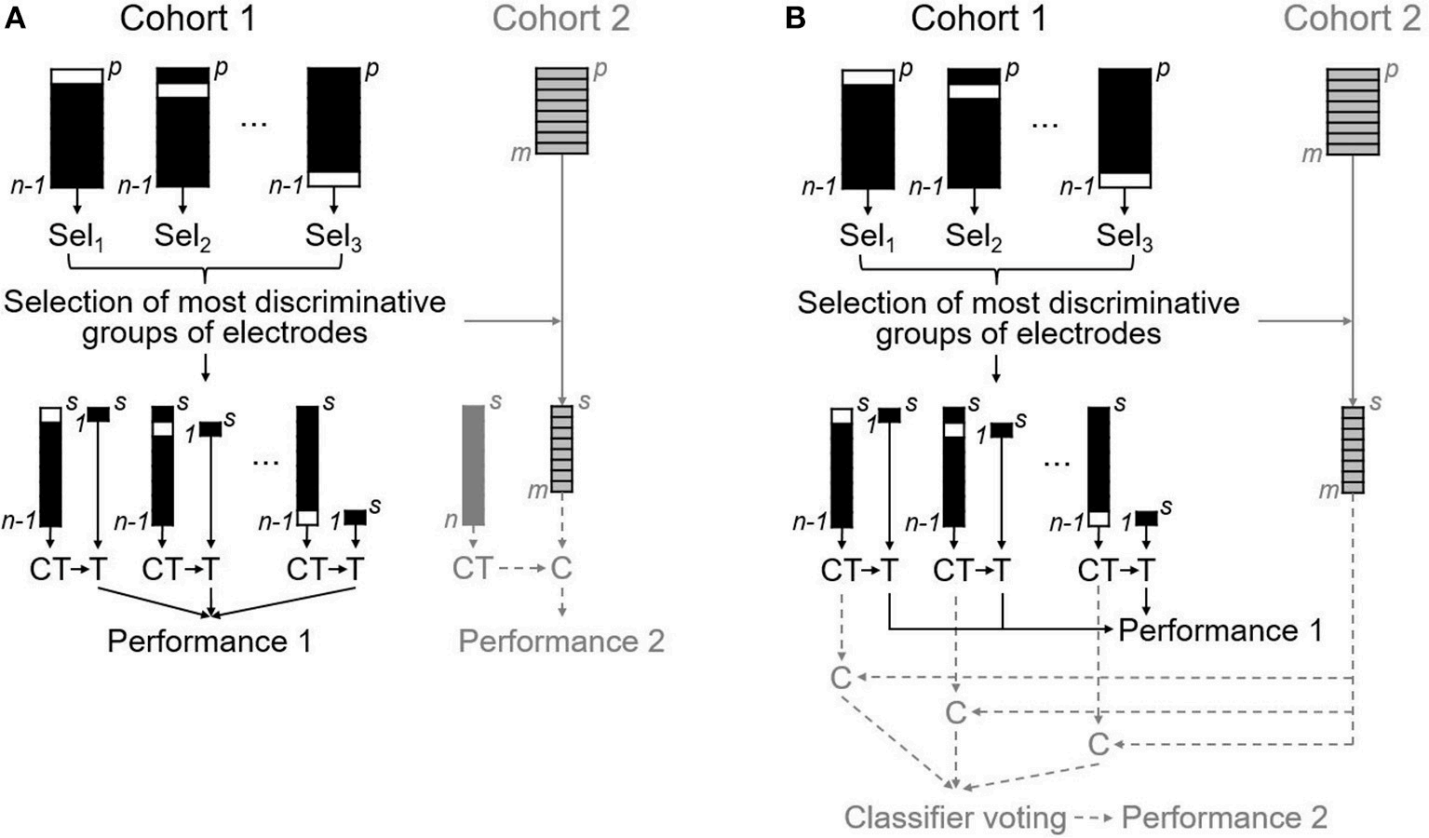
The statistical model for prediction of VNS efficacy based on EEG analysis. Schematics representing the development of the statistical model on data from Cohort 1 outlining the selection of the most discriminative electrodes groups as well as classifier training (CT) and testing (T) performed in LOO manner (the black schematics); and two approaches taken for the validation procedure, whereby our statistical model was applied to an independent data-set from Cohort 2 (the gray schematics). Equivalent steps taken in each of the two validation approaches are visualized using solid lines, including the reduction of Cohort 2 by selecting the maximally discriminative electrodes identified in Cohort 1. The two approaches differ in the steps represented by dashed lines—i.e., the classification (C) of the reduced dataset using: **(A)** a single classifier trained on all *n* subjects from Cohort 1, and **(B)** voting of *n* classifiers trained on *n*-1 subsets of Cohort 1.

The statistical model for prediction of VNS efficacy was developed from data obtained in Cohort 1 in the following steps: Firstly, electrodes were grouped into seven anatomical regions as follows: (1) left frontal—Fp1, F3, Fz; (2) right frontal—Fp2, F4, Fz; (3) left anterotemporal—F7, T3; (4) right anterotemporal—F8, T4; (5) central—C3, Cz, C4; (6) left posterior quadrant—P3, Pz, T5, O1; (7) right posterior quadrant—P4, Pz, T6, O2. Specifically, mean values were calculated for the respective groups of electrodes. This resulted in 196 electrode group variables (7 conditions × 4 frequency bands × 7 anatomical regions). In the second step, the maximally discriminative electrode group variables were then selected using stepwise logistic regression performed in leave-one-out (LOO) manner; specifically, with *n* patients, logistic regression was performed over *n* iterations, each with one subject omitted. This approach allowed us to avoid overestimating the classification results, which occurs when classification is performed on electrode groups selected using all subjects simultaneously. Thirdly, electrode group variables identified most frequently as maximally discriminative after the *n* LOO iterations were used for classification using three classifiers; namely, logistic regression (LR), linear support vector machines (SVM), and linear discriminant analysis (LDA). In this step, we used a LOO cross-validation to split the data into training and testing sets: one patient was chosen randomly as a testing subject and the remaining patients were employed for training the classifier. The testing subject was then classified as belonging to the Responder or to the Non-responder class, and the resulting class label was compared with the true classification label. This procedure was repeated using each of the subjects as the testing subject sequentially, and the overall classification performance measures of accuracy, sensitivity, and specificity were calculated (see the black schematics in Figure 2). Fourth, using one-sample binomial test we performed a comparison of the achieved classification accuracies with a classification by chance. We also attempted to predict VNS efficacy based on 532 possible single electrode variables (7 conditions × 4 frequency bands × 19 electrodes; see Supplementary Material 1).

#### Validation of the Statistical Model

The validity of the aforementioned statistical model was verified on the independent data-set obtained from Cohort 2. This validation cohort consisted of patients suffering from drug-resistant epilepsy who were implanted with VNS in our Center between 2013 and 2015. We obtained EEG data recorded using an identical EEG system and pre-surgical assessment protocol, and processed mathematically in the exact same way described above. Patients were classified as Responders and Non-responders with our statistical model using two approaches (see the gray schematics in Figure 2). Both began with data reduction, whereby we selected only data corresponding to the maximally discriminative electrode group variables identified based on Cohort 1. In the first approach, this data reduction was followed subsequently by a classification of *m* subjects from Cohort 2 as Responders or Non-responders based on a single classifier, which was trained on all *n* subjects from the Cohort 1. In contrast, in the second approach we classified the reduced data of Cohort 2 using majority voting of *n* classifiers trained on *n*-1 subsets of Cohort 1. The resulting indices for accuracy, sensitivity, and specificity were then compared to those from the classification of Cohort 1.

## Results

### Participants

#### Cohort 1

The VNS device was implanted in 110 patients in our center between 2005 and 2012. We excluded 50 patients (45%) from further analyses−17 (15%) because of poor-quality pre-surgical EEG, 13 (12%) due to attrition, and 20 (18%) who switched between VNS outcomes during the follow-up period (see Supplementary Material 2 for details). Demographics for the 60 patients included in the analyses are summarized in Table 1. According to the response to VNS, patients were subdivided into 35 (58%) Responders and 25 (42%) Non-responders. When examining demographic data, we observed significant differences between Responders and Non-responders in patients' age at epilepsy onset, duration of epilepsy before VNS implantation, and treatment by valproic acid (Table 1).

**Table 1:** Demographic and treatment data for Cohort 1 and Cohort 2.

		**Cohort 1**				**Cohort 2**			
		**Combined (*n* = 60)**	**Non-responders (*n* = 25)**	**Responders (*n* = 35)**	***p***	**Combined (*n* = 22)**	**Non-responders (*n* = 10)**	**Responders (*n* = 12)**	***p***
Type of epilepsy, *n* (%)	TLE	14 (23)	6 (24)	8 (23)	1.000	6 (27)	3 (30)	3 (25)	0.801
	Extra-TLE	43 (72)	18 (72)	25 (72)		15 (68)	6 (60)	9 (75)	
	IGE	3 (5)	1 (4)	2 (6)		1 (5)	1 (10)	0 (0)	
Gender, *n* (%)	Females	34 (57)	17 (68)	17 (49)	0.188	11 (50)	4 (40)	7 (58)	0.670
	Males	26 (43)	8 (32)	18 (51)		11 (50)	6 (60)	5 (42)	
Age (years) at VNS implantation (median, min-max)		33 (15–65)	30 (15–65)	36 (18–63)	0.134	31 (22–71)	26 (22–42)	40 (22–71)	0.069
Age (years) at epilepsy onset (median, min-max)		9 (1–51)	13 (1–27)	6 (1–51)	0.014	10 (0–59)	8 (0–18)	12 (4–59)	0.123
Duration (years) of epilepsy before vagal nerve stimulator implantation (median, min-max)		22 (4–60)	15 (4–55)	26 (7–60)	0.019	20 (2–49)	20 (14–34)	19 (2–49)	0.872
Duration (years) of VNS (median, min-max)		6 (3–11)	6 (3–10)	6 (3–11)	0.581	3 (2–3)	3 (2–3)	3 (2–3)	0.628
Treatment at the time of VNS implantation, *n* (%)	BRV	2 (3)	2 (8)	0 (0)	0.169				
	CBZ	32 (53)	15 (60)	17 (49)	0.439	9 (41)	3 (30)	6 (50)	0.415
	CLB	1 (2)	1 (4)	0 (0)	0.417				
	CLZ	13 (22)	6 (24)	7 (20)	0.758	4 (18)	3 (30)	1 (8)	0.293
	ESL	3 (5)	2 (8)	1 (3)	0.565	5 (23)	4 (40)	1 (8)	0.135
	GBP	1 (2)	0 (0)	1 (3)	1.000				
	LCM	6 (10)	1 (4)	5 (14)	0.386	9 (41)	6 (60)	3 (25)	0.192
	LEV	36 (60)	16 (64)	20 (57)	0.790	10 (45)	4 (40)	6 (50)	0.691
	LTG	27 (45)	11 (44)	16 (46)	1.000	9 (41)	4 (40)	5 (42)	1.000
	PGB	5 (8)	2 (8)	3 (9)	1.000	1 (5)	0 (0)	1 (8)	1.000
	PHE	1 (2)	0 (0)	1 (3)	1.000				
	PHT	4 (7)	1 (4)	3 (9)	0.634	1 (5)	1 (10)	0 (0)	0.455
	PRM	3 (5)	1 (4)	2 (6)	1.000	2 (9)	1 (10)	1 (8)	1.000
	TPM	13 (22)	5 (20)	8 (23)	1.000	2 (9)	1 (10)	1 (8)	1.000
	VPA	14 (23)	2 (8)	12 (34)	0.028	5 (23)	3 (30)	2 (17)	0.624
	ZNS	8 (13)	5 (20)	3 (9)	0.259	9 (41)	4 (40)	5 (42)	1.000
Number of AEDs used at the time of VNS implantation, *n* (%)	1	4 (7)	1 (4)	3 (9)	0.974	1 (5)	0 (0)	1 (8)	0.495
	2	17 (28)	8 (32)	9 (26)		7 (32)	3 (30)	4 (33)	
	3	26 (43)	11 (44)	15 (43)		7 (32)	2 (20)	5 (42)	
	4	12 (20)	5 (20)	7 (20)		5 (23)	3 (30)	2 (17)	
	5	1 (2)	0 (0)	1 (3)		2 (9)	2 (20)	0 (0)	

*AEDs, antiepileptic drugs; BRV, brivaracetam; CBZ, carbamazepine; CLB, clobazam; CLZ, clonazepam; ESL, eslicarbazepine; Extra-TLE, extratemporal lobe epilepsy; GBP, gabapentin; LCM, lacosamide; LEV- levetiracetam; LTG, lamotrigine; NA, not applicable; PGB, pregabalin; PHE, phenobarbital; PHT, phenytoin; PRM, primidone; TLE, temporal lobe epilepsy; TPM, topiramate; VNS, vagal nerve stimulation; VPA, valproic acid; ZNS, zonisamide*.

#### Cohort 2

In total, the VNS device was implanted in 25 patients between 2013 and 2015. Three patients were excluded from our analyses due to poor-quality pre-surgical EEG recordings. Data from the remaining 22 patients were used for independent validation: 12 (54.5%) Responders and 10 (45.5%) Non-responders. The demographic data are summarized in Table 1. The Cohort 2 did not differ from the Cohort 1 in the demographic characteristics, apart from the anticipated difference in duration of VNS (*p* < 0.001) and subtle differences in antiepileptic treatment (specifically, eslicarbazepine, lacosamide, and zonisamide were more frequent in Cohort 2; *p* = 0.029, *p* = 0.003, and *p* = 0.012, respectively).

### RPW Differences Between Responders and Non-responders

**Figure 3:**
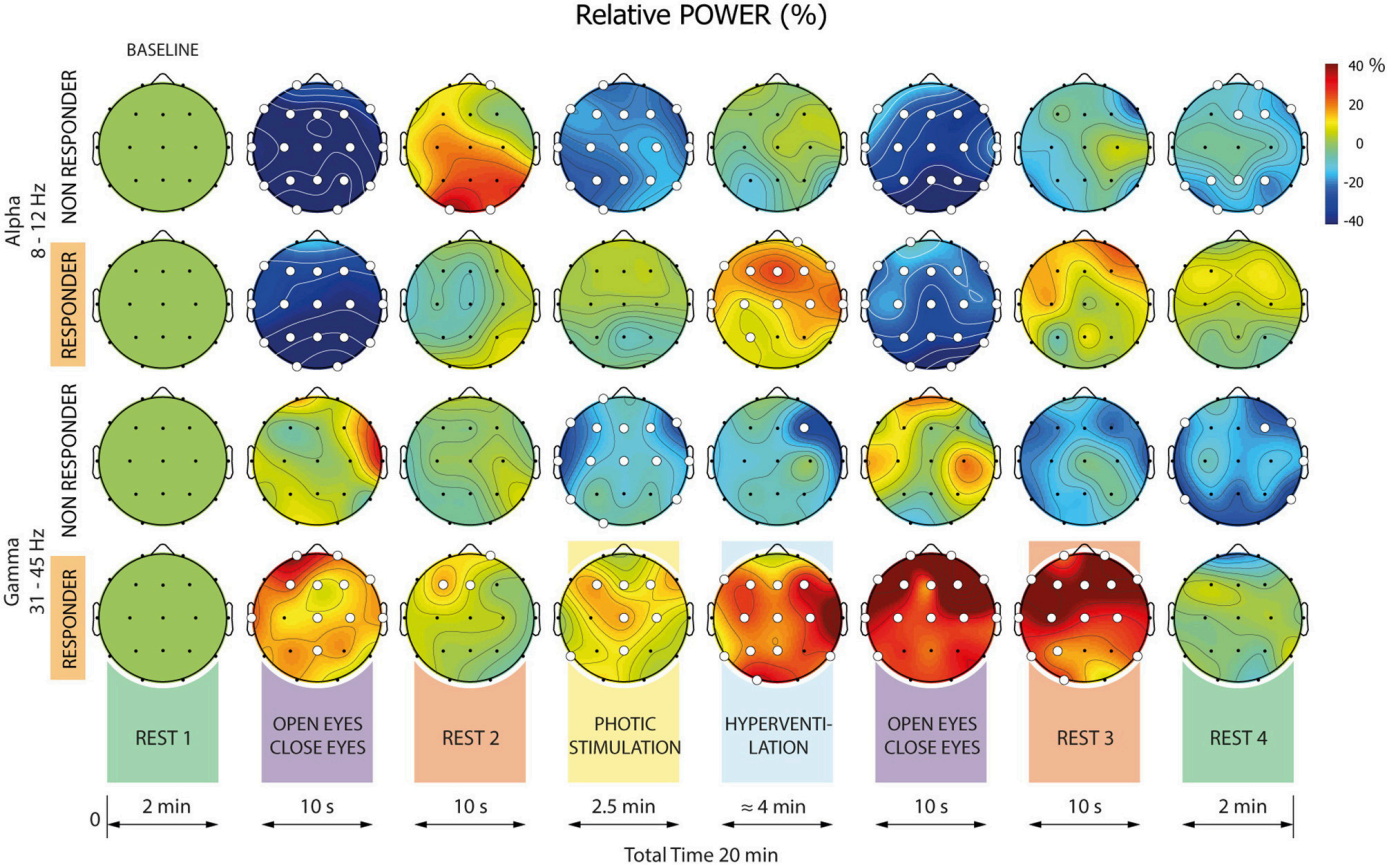
Relative mean powers (RPWs) for all frequency bands. Each head represents RPW in a given condition and frequency band. The RPW percentage scale is displayed on the right-hand side. Where a statistically significant difference exists in a given condition relative to baseline (Rest#1), the electrode is marked by a white dot. If no significant difference exists, the electrode is marked by a black dot.

**Figure 4:**
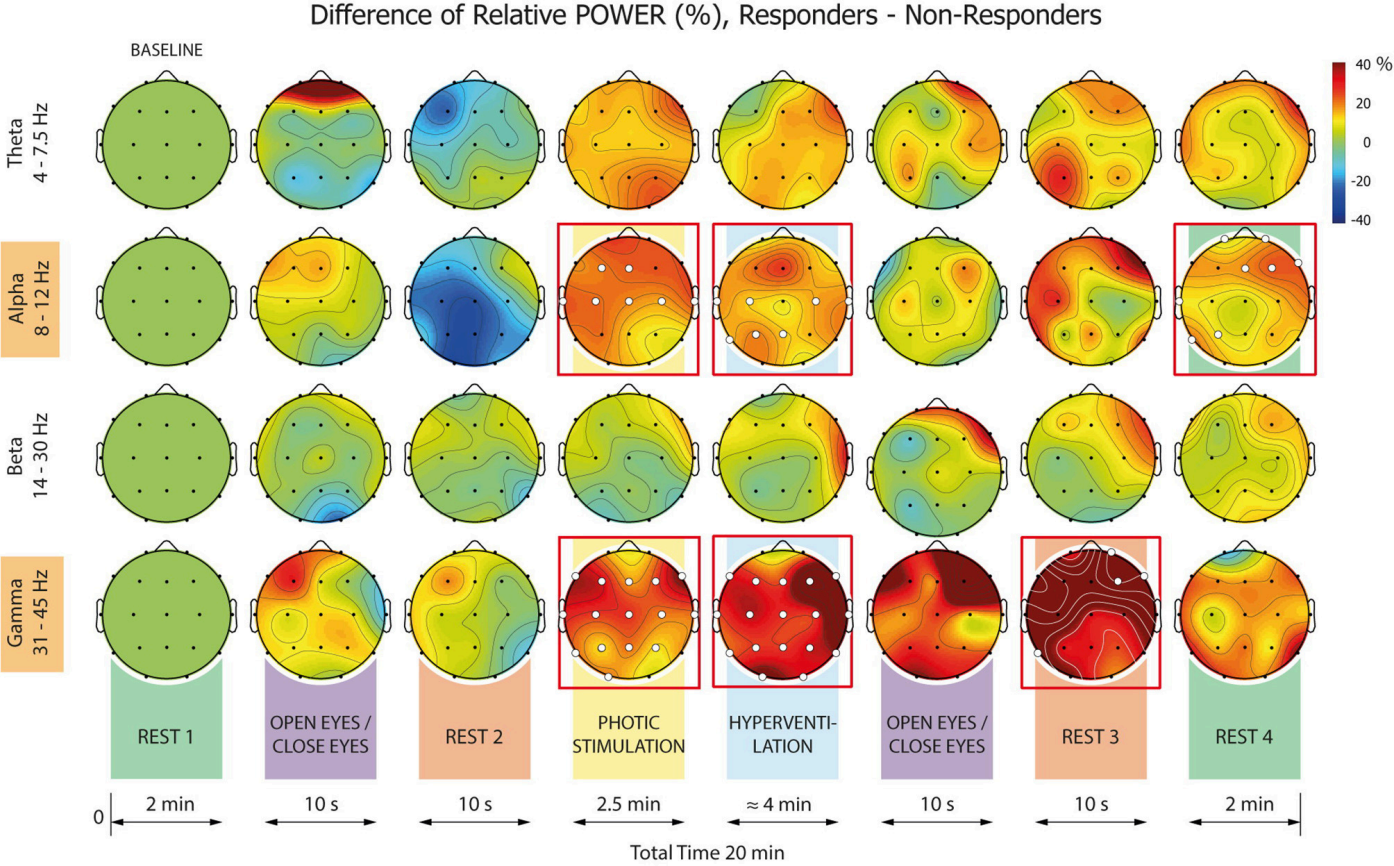
Relative mean power (RPW) differences between Responders and Non-responders. Statistically significant or non-significant differences in a given electrode are marked by a white or black dot, respectively. The significant differences between Responders and Non-responders were identified in alpha and gamma frequency ranges only (heads in red boxes).

In Cohort 1, computation of RPWs and their dynamics in the recording sites revealed significant changes for both Responders and Non-responders in all investigated frequency bands, all defined conditions, and the majority of electrodes (Figure 3). These changes were largely equivalent for both Responders and Non-responders within specific frequency bands and conditions. In the alpha and gamma frequency bands, however, there were striking dissimilarities between the two patient groups in conditions, with the most prominent differences during photic stimulation (PS) and hyperventilation (HV). Comparing Responders and Non-responders with respect to the RPW in all pre-defined frequency bands and conditions, we revealed significant differences in alpha and gamma frequency bands (Figure 4).

When analyzing the alpha band, significant differences between Responders and Non-responders were found in the following conditions: 4-PS, 5-HV, and 8–Rest#4. The differences were present over different brain regions in each condition: In 4-PS, there were differences over central and anterior areas (absence of desynchronization in Responders); in the 5-HV interval we observed differences over central and posterior regions (significantly higher synchronization in Responders); and in 8-R, differences were localized to right anterior and left posterior areas (higher power in Responders but persisting desynchronization in Non-responders).

When focusing on the gamma frequency band, we observed significant differences (gamma synchronization in Responders and desynchronization in Non-responders) in RPW within 4-PS, 5-HV and 7-Rest#3: In the first two conditions, differences were distributed across almost the whole scalp. The differences in RPWs within 7-Rest#3 were observed within right anterior and left posterior areas.

### Prediction of VNS Response—Statistical Model

**Figure 5:**
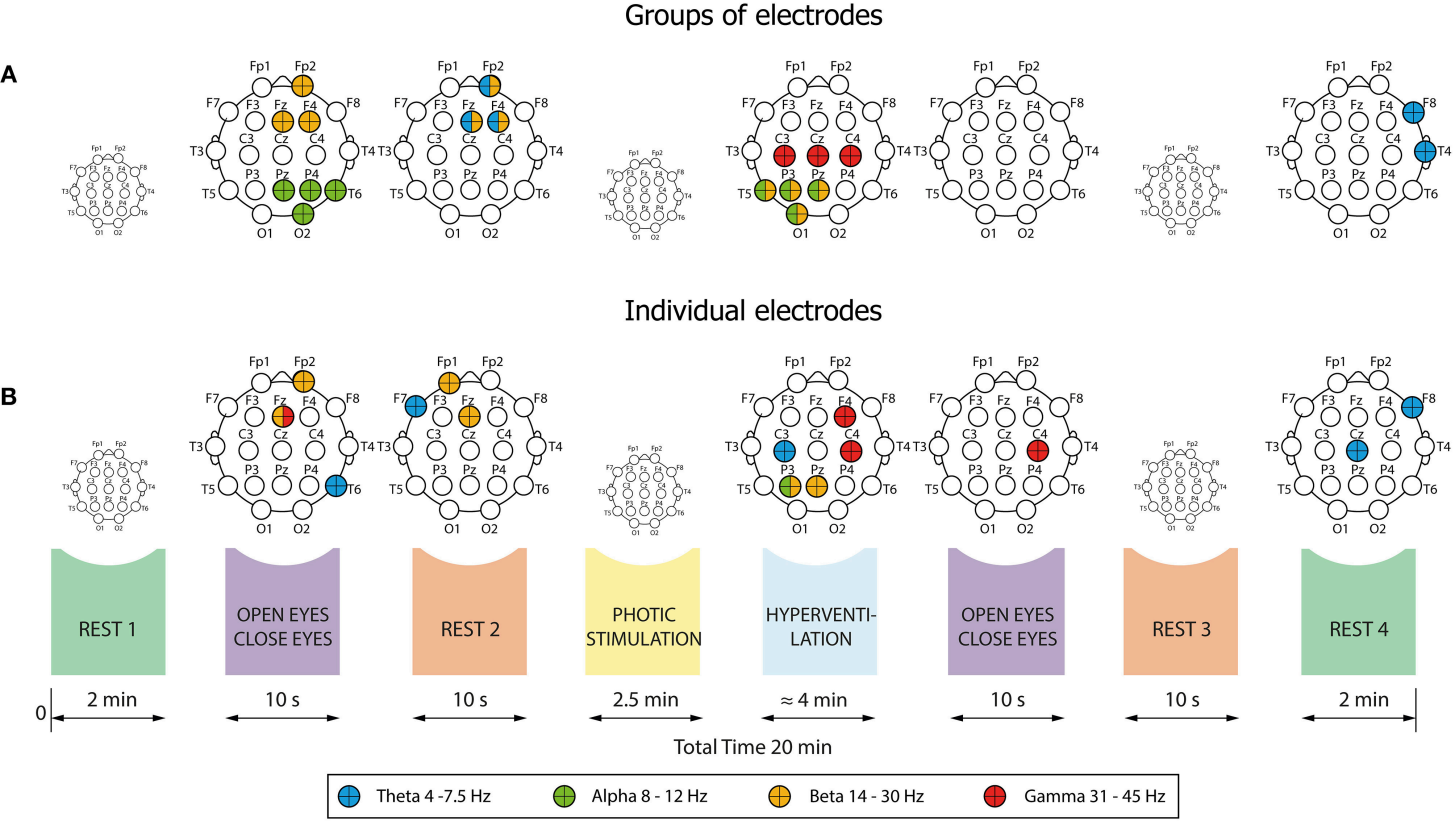
Electrode groups and individual electrodes selected for statistical modeling. Electrode groups **(A)** and individual electrodes **(B)** selected for the development of the statistical model, in each condition and frequency band.

**Figure 6:**
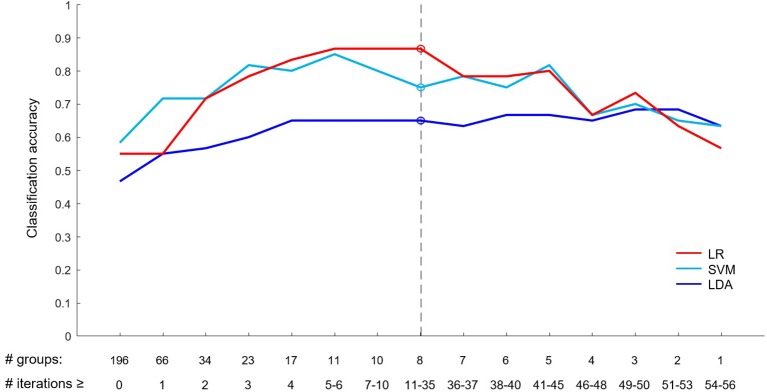
Classification accuracy based on a number of selected groups of electrodes. The bottom row of numbers shows a minimum number of LOO iterations (# iterations) in which a given number of electrode groups (# groups) were selected as most discriminative (e.g., eight electrode groups were selected as maximally discriminative in 35 or more LOO iterations). The best LR classification accuracy and corresponding results for SMV and LDA (shown in Table 2) are depicted by circles and a dashed line.

Based on the EEG data of Cohort 1, the statistical model was developed. Eight groups of electrodes were selected as the most discriminative in this statistical model (visualized in Figure 5). The best classification results based on these eight most discriminative groups of electrodes were obtained using the LR classifier, achieving 86.7% accuracy, with 88.6% sensitivity and 84% specificity (Table 2). This classification accuracy was significantly higher than those achieved by chance (*p* < 0.001). The SVM classifier achieved lower accuracy (75%) but it was still significantly higher than that achieved by chance (*p* = 0.004). The lowest classification performance measures were obtained in classification using LDA (accuracy 65%). Detailed visualizations of classification accuracy are provided in Figure 6. The final statistical model is described in more detail in Supplementary Material 3.

**Table 2:** Classification performance indices for electrode group variables.

	**Cohort 1−60 patients**				**Cohort 2−22 patients**			
	**Accuracy**	**Sensitivity**	**Specificity**	***p***	**Accuracy**	**Sensitivity**	**Specificity**	***p***
LR	86.7	88.6	84.0	<0.001	86.4	83.3	90.0	0.006
SVM	75.0	62.9	92.0	0.004	77.3	58.3	100.0	0.043
LDA	65.0	48.6	88.0	0.147	45.5	16.7	80.0	0.879

*LDA, linear discriminant analysis; LR, logistic regression; p, p-value calculated using one-sample binominal test; SVM, linear support vector machines*.

### Validation of the Statistical Model

The data of patients in Cohort 2 were used for independent validation. When comparing the results of our classification model applied to Cohort 2 against real-life outcome, the best results were again obtained for the model using the LR classifier achieving an accuracy of 86.4%, sensitivity of 83.3%, and specificity of 90%. The complete results achieved using classifier voting are summarized in Table 2. Classification performance based on a single classifier was lower (data not shown).

## Discussion

Over 80,000 epilepsy patients have been treated worldwide using vagal nerve stimulation (VNS), a standard of modern non-pharmacological treatment. Importantly, the number of epileptic patients who are eligible for VNS therapy is approximately three million subjects worldwide. In this light, a method for the reliable prediction of individual efficacy of VNS therapy is desperately needed.

Recently, several authors have attempted to identify predictors of VNS outcome (1, 2, 4, 13). Despite these efforts, however, clinical predictors of individual responsiveness to VNS therapy remain elusive. Similar estimates of efficacy are reported for diverse neurostimulation techniques in the treatment of drug-resistant epilepsy (i.e., VNS, Deep Brain Stimulation of Anterior Thalamic Nuclei, Brain Responsive RNS System, transcutaneous VNS, or external Trigeminal Nerve Stimulation), which might suggest a more consequential impact of external stimuli *per se* on epileptic activity. For this reason, we retrospectively evaluated routine EEG data acquired before implantation in a large cohort of VNS patients. Using standard computations of power spectral analyses of interictal EEG, we reveal significant differences between responders and non-responders in two pre-defined frequency bands (alpha and gamma) and four conditions of standard clinical assessment. Based on RPWs and their dynamics, we have developed and validated a statistical model for prediction of VNS efficacy that discriminated between responders and non-responders with almost 90% accuracy.

Our primary finding is that VNS responders and non-responders differ significantly in EEG power dynamics within alpha and gamma frequency bands prior to therapy. Whilst both patient groups demonstrated equivalent alpha desynchronization during eyes opening, they differed in alpha reactivity to photic stimulation and hyperventilation; specifically, responders showed no decrease in alpha power during the former but an enormous increase during the latter. This reactivity pattern stands in contrast to that observed in healthy individuals, in whom photic stimulation typically leads to alpha attenuation and standardized hyperventilation has been shown to decrease alpha power (14). Interestingly, we also observed significant increases in gamma power during both photic stimulation and hyperventilation in responders relative to non-responders. Hyperventilation-induced physiological changes are thought to be a consequence of increased neuronal excitability resulting from the hypocapnia-induced alkalosis (15, 16). Significantly enhanced alpha and gamma activities during hyperventilation in responders might reflect distinct properties of responders' brains vis-a-vis neuronal excitability and synaptic transmission. Nevertheless, hyperventilation is a complicated practice which, in epileptic subjects, results in unpredictable responses. The reasons for this remain unclear (e.g., metabolic hypersynchronization, altered neurotransmitters, etc.). It also remains unclear why the alpha (and less expressed gamma) desynchronization persist in our non-responders at the end of EEG recordings (Rest#4). It seems that the protocol itself, particularly photic stimulation and hyperventilation, induces some change from the resting-state baseline for non-responders. Differential dynamics of power changes after the stimulation—faster in responders and slower in non-responders—might represent another characteristic in which these groups of patients differ substantially.

Interestingly, valproic acid (VPA) was used more frequently in pharmacological treatment prior to implantation in our VNS responders compared to the non-responders. We might therefore ask whether differences in EEG reactivity observed in our study is related to some pharmacological imprint. Although this speculative explanation cannot be fully excluded, it seems unlikely that pharmacological impact on EEG is the main factor driving our findings. Still more unlikely is substantial VPA impact on the prediction of VNS efficacy, bearing in mind that only one third of responders were treated with VPA and the accuracy of individual VNS efficacy prediction was almost 90%.

Since Hans Berger's initial observation in 1933, the best known example of EEG reactivity is alpha attenuation (alpha blocking and desynchronization). This is observed typically when subjects open their eyes (the Berger effect), but alpha also disappears when subjects become drowsy and it can be blocked by numerous kinds of external stimuli (visual or auditory) or mental operations (e.g., imagery, visualization, mental arithmetics). This implies that alpha reactivity as a modality-independent, general phenomenon, reflecting the functional modes of thalamo-cortical and cortico-cortical loops that facilitate/inhibit the transmision of information in the brain (17, 18). Particularly noteworthy is the high inter-individual variability in alpha reactivity; it has been shown to differ between extraverts and introverts, for example, and there is less pronounced alpha desynchronization in people with high intelligent quotient during several cognitive tasks (19, 20). Alpha reactivity has also been reported to decrease with aging (21–24) and in patients with mild cognitive impairment and Alzheimer disease (25), and a lack of alpha reactivity has been used to predict the long-term deterioration of higher functions in subjects with cognitive decline (26). From this viewpoint, our discovery of differential alpha reactivity in VNS responders vs. non-responders further emphasizes the multifold functions of the diffuse alpha system (27).

In our study, significant differences in EEG power dynamics within the gamma frequency band between VNS responders and non-responders very likely reflects distinct reactivity of true brain gamma. When interpreting thoroughly our results in this frequency band, however we shall keep in mind the study of Whitham et al. (28); these authors showed recently that even the normal resting EEG might reveal significant contamination with electromyography (EMG) activity in this frequency band. Further, the level of EMG contamination increased dramatically when subjects perform various experimental tasks and are sitting during EEG recordings (29). More recently, however, Boytsova et al. showed that EMG contamination does not necessarily hide high-frequency EEG and does not preclude qualitative detections of electroencephalographic correlates of mental activities in beta and low gamma frequency ranges (30). Unfortunately, despite the availability of several techniques for the reduction of muscular artifacts from EEG traces, at a present none are able to guarantee that analyzed data are completely free of high-frequency artifacts (29). In our study, EEG was recorded under standard conditions: patients laid comfortably and were instructed to relax (including their facial muscles), no task was performed, and recordings from patients containing artifacts identified with careful visual inspection (e.g., muscular activity) were excluded (17 out of 110 patients). As such, we strongly believe an increased gamma power during both photic stimulation and hyperventilation in responders relative to non-responders is not resulting from distinct muscle artifacts contaminations.

Both brain rhythms—alpha and gamma—are considered to represent a kind of universal code consistent with their putative role in brain signaling (27). Both are generated in a widely distributed system, with a major role of thalamocortical circuits in their origin (31–33). The differential impact of external stimuli on alpha and gamma between VNS responders and non-responders might be mediated by differences in neuronal interconnectivity and different levels of neurotransmitters within underlying cerebral matrices (24). Such differences might influence the effect of external stimuli delivered to thalamocortical circuits and other brain networks via the vagal nerve (34, 35). Consistent with this notion, many consider the mechanism of VNS action to be modulation of synaptic activity in the thalamus and thalamocortical projections, increased plasticity in GABA receptors, and modulation of GABAergic activity that is related directly to gamma oscillations (6, 36). Indeed, one of the most plausible factors involved in gamma variation between our populations of responders vs. non-responders is represented by the role of GABA neurotransmission and divergent dynamics of inhibitory interneuron networks within the central nervous system (37, 38). The close relationship between different types of external stimulation and brain reactivity we have observed might be indicative of a common mechanism underlying the various forms of neurostimulation in epilepsy treatment.

Finally, as seen in the developed and validated statistical model, for accurate prediction of VNS efficacy distinct recording electrode groups, different conditions (especially hyperventilation, but also eyes opening/closing and resting periods), and all frequency bands were selected as the most discriminative ones. Using our approach we achieved high accuracy, sensitivity and specificity in both well-defined and predominant VNS patients. We also evaluated a statistical model based on single electrodes, which had slightly superior classification performance in Cohort 1 and predominant VNS patients but failed in the independent validation set of Cohort 2. This indicates that groups of electrodes are better suited for VNS efficacy prediction—by integrating data from larger regions, such groupings appear to produce more robust results.

To conclude, we have revealed that EEG reactivity to external stimuli used during routine pre-operative EEG investigation differs between VNS responders and non-responders. Moreover, we have developed and validated a statistical tool that can predict with extremely high accuracy whether or not individual drug-resistant epileptic patients will benefit from VNS treatment (Patent Number EP3437692-A1). This electrophysiological marker could prove invaluable when providing patients with expected postoperative prognosis. Further research is required before this can be achieved, however; our findings come from a retrospective and monocenter study, with a limited number of patients comprising the independent validation. Our results must therefore be replicated in prospective, multicentre, and well-designed clinical study in order to obtain a clear-cut statistical power.

## Ethics Statement

Ethic committee in St. Anne's University hospital approved this study. All patients gave their informed consent.

## Author Contributions

MB, RR, PJ, JChr, and DS: identification of research topic, preparation of study design, interpretation of results. ID and MP: preparation of manuscript, design of study, interpretation of results. JChl and PJ: mathematical analysis. EK: statistical analysis.

### Conflict of Interest Statement

The patent application was applied (patent applicant Masaryk University; inventors are the authors of the manuscript: MB, ID, EK, JChl, RR, JChr, MP). The remaining authors declare that the research was conducted in the absence of any commercial or financial relationships that could be construed as a potential conflict of interest.
