# Supplementary material for: EEG Reactivity Predicts Individual Efficacy of Vagal Nerve Stimulation in Intractable Epileptics
Source: Front Neurol. 2019 May 2;10:392. doi: 10.3389/fneur.2019.00392 (PMC6507513; doi:10.3389/fneur.2019.00392)

Supplementary Material

**Supplementary Material 1: Statistical model based on single electrode variables**

In a parallel, we attempted to develop a statistical model for prediction of VNS efficacy based on single electrode variables. This model was based on 532 possible “electrode” variables (7 conditions x 4 frequency bands x 19 electrodes) and was analogous to the model described in the main text. The 16 most discriminative electrodes selected for this type of statistical model are shown in Figure 5.

This statistical model working single electrode variables had even higher accuracy than the model based on electrode groups variables in Cohort 1. Logistic regression (LR) classifier based or the 16 single electrode variables had accuracy of 90%, with 94.3% sensitivity and 84% specificity (Supplementary Table 1.1). Besides, same accuracy (90%) but different sensitivity (88.6%) and specificity (92%) was achieved also using LR classifier based on 13 single electrode variables. Detailed visualizations of classification accuracies are provided in Supplementary Figure 1.1.

When retesting the accuracy of this model based on individual electrodes in Cohort 2, i.e. in independent data set, the model lost its predictive value (Supplementary Table 1.1). The highest accuracy was only about 50% for LR and LDA classifiers in both 13 and 16 single electrode models.

**Supplementary Table 1.1: Classification performance for single electrode variables**

|  | Cohort 1 – 60 patients | | | | Cohort 2 – 22 patients | | | |
| --- | --- | --- | --- | --- | --- | --- | --- | --- |
|  | accuracy | sensitivity | specificity | p | accuracy | sensitivity | specificity | p |
| **16 single electrode variables** | | | | | | | | |
| LR | 90.0 | 94.3 | 84.0 | <0.001 | 45.5 | 50.0 | 40.0 | 0.879 |
| SVM | 85.0 | 85.7 | 84.0 | <0.001 | 36.4 | 33.3 | 40.0 | 0.977 |
| LDA | 68.3 | 54.3 | 88.0 | 0.058 | 50.0 | 25.0 | 80.0 | 0.775 |
| **13 single electrode variables** | | | | | | | | |
| LR | 90.0 | 88.6 | 92.0 | <0.001 | 50.0 | 50.0 | 50.0 | 0.775 |
| SVM | 83.3 | 85.7 | 80.0 | <0.001 | 45.5 | 50.0 | 40.0 | 0.879 |
| LDA | 70.0 | 54.3 | 92.0 | 0.033 | 59.1 | 41.7 | 80.0 | 0.473 |

LDA – linear discriminant analysis, LR – logistic regression, p – p-value calculated using one-sample binominal test, SVM – linear support vector machines

**Supplementary Figure 1.1:** **Classification accuracy based on the number of selected single electrode variables.**

The bottom row of numbers shows a minimum number of LOO iterations (# iterations) in which a given number of electrodes (# electrodes) were selected as maximally discriminative (e.g., 13 single electrodes were selected as maximally discriminative in 11 or more LOO iterations). The best LR classification accuracies and corresponding results for SMV and LDA (shown in Supplementary Table 1.1) are depicted by circles and dashed lines.

**
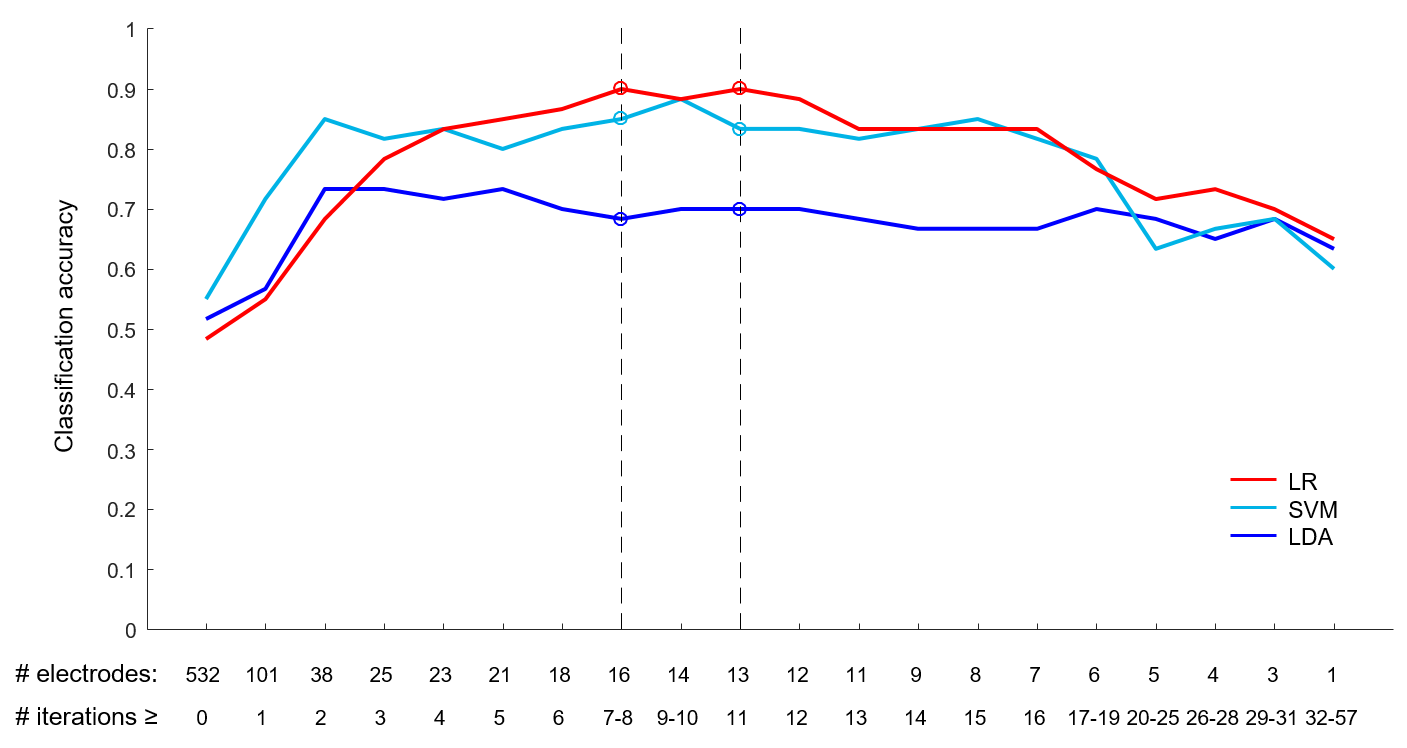
**

**Supplementary Material 2: Classification of patients who switched their response to VNS stimulation during follow-up period**

We excluded 20 patients in Cohort 1 from the main classification because they exhibited critical changes in their efficacy to VNS during follow-up. These patients were classified subsequently according to their predominant response; namely, the response presented for more than half of the follow-up period. Nine (45%) of these patients were classified as Predominant Responders, the remaining 11 (55%) patients were classified as Predominant Non-responders. Demographic data did not differ between these two patients groups, neither type nor number of AEDs (Supplementary Table 2.1).

We tested our models developed using data of Responders and Non-responders from Cohort 1 in this group of Predominant Responders and Predominant Non-responders too. The prediction accuracy was 80.0% (77.8% sensitivity, 81.8% specificity) while using 13 or 16 single electrode variables and the single LR classifier as well as the classifier voting. When modeled as grouped electrodes, the classification performance was somewhat lower; specifically, 75.0% accuracy (77.8% sensitivity, 72.7% specificity) with the single LR classifier and 65.0% accuracy (77.8% sensitivity, 54.5% specificity) using the classifier voting. The results are summarized in Supplementary Table 2.2.

**Supplementary Table 2.1: Demographic and treatment data for Predominant Responders and Predominant Non-responders.**

|  |  | **Predominant Responders and Predominant Non-responders (n=20)** | **Predominant Responders vs. predominant Non-responders** | | |
| --- | --- | --- | --- | --- | --- |
|  |  |  | **Predominant Non-responders**  **(n=11)** | **Predominant Responders**  **(n=9)** | **p** |
| Type of epilepsy, n (%) | TLE | 6 (30) | 5 (46) | 1 (11) | 0.157 |
|  | Extra-TLE | 14 (70) | 6 (55) | 8 (89) |  |
|  | IGE | 0 (0) |  |  |  |
| Gender, n (%) | Females | 13 (65) | 8 (73) | 5 (56) | 0.642 |
|  | Males | 7 (35) | 3 (27) | 4 (44) |  |
| Age (years) at VNS implantation (median, min-max) | | 40 (21-49) | 39 (21-47) | 40 (26-49) | 0.603 |
| Age (years) at epilepsy onset (median, min-max) | | 9  (1-28) | 10  (1-28) | 8  (1-16) | 0.552 |
| Duration (years) of epilepsy before vagal nerve stimulator implantation (median, min-max) | | 28  (4-42) | 27  (4-42) | 29  (11-42) | 0.656 |
| Duration (years) of VNS (median, min-max) | | 6  (2-9) | 6  (2-9) | 6  (2-8) | 0.941 |
| Treatment at the time of VNS implantation, n (%) | CBZ | 11 (55) | 4 (36) | 7 (78) | 0.092 |
|  | CLZ | 4 (20) | 1 (9) | 3 (33) | 0.285 |
|  | LCM | 1 (5) | 1 (9) | 0 (0) | 1.000 |
|  | LEV | 7 (35) | 4 (36) | 3 (33) | 1.000 |
|  | LTG | 11 (55) | 7 (64) | 4 (44) | 0.653 |
|  | PGB | 6 (30) | 1 (9) | 5 (56) | 0.050 |
|  | PRM | 4 (20) | 2 (18) | 2 (22) | 1.000 |
|  | TPM | 3 (15) | 3 (27) | 0 (0) | 0.218 |
|  | VPA | 3 (15) | 1 (9) | 2 (22) | 0.566 |
|  | ZNS | 6 (30) | 3 (27) | 3 (33) | 1.000 |
| Number of AEDs used at the time of VNS implantation, n (%) | 1 | 1 (5) | 1 (9) | 0 (0) | 0.130 |
|  | 2 | 5 (25) | 4 (36) | 1 (11) |  |
|  | 3 | 11 (55) | 6 (55) | 5 (56) |  |
|  | 4 | 3 (15) | 0 (0) | 3 (33) |  |
|  | 5 | 0 (0) | 0 (0) | 0 (0) |  |

AEDs – antiepileptic drugs, CBZ – carbamazepine, CLZ – clonazepam, Extra-TLE – extratemporal lobe epilepsy, LCM – lacosamide, LEV- levetiracetam, LTG – lamotrigine, PGB – pregabalin, PRM – primidone, TLE – temporal lobe epilepsy, TPM – topiramate, VNS – vagal nerve stimulation, VPA – valproic acid, ZNS – zonisamide

**Supplementary Table 2.2**: C**lassification performance for Predominant Responders vs. Predominant Non-Responders**

|  | **Statistical model – electrode group variables** | | | | **Statistical model – single electrode variables** | | | | | | | |
| --- | --- | --- | --- | --- | --- | --- | --- | --- | --- | --- | --- | --- |
|  |  |  |  |  | **13 single electrode variables** | | | | **16 single electrode variables** | | | |
|  | accuracy | sensitivity | specificity | p | accuracy | sensitivity | specificity | p | accuracy | sensitivity | specificity | p |
| Classification using a single classifier | | | | | | | | | | | | |
| LR | 75.0 | 77.8 | 72.7 | 0.065 | 80.0 | 77.8 | 81.8 | 0.025 | 80.0 | 77.8 | 81.8 | 0.025 |
| SVM | 75.0 | 66.7 | 81.8 | 0.065 | 85.0 | 88.9 | 81.8 | 0.008 | 85.0 | 88.9 | 81.8 | 0.008 |
| LDA | 60.0 | 44.4 | 72.7 | 0.440 | 65.0 | 44.4 | 81.8 | 0.273 | 70.0 | 44.4 | 90.9 | 0.145 |
| Classification using classifier voting | | | | | | | | | | | | |
| LR | 65.0 | 77.8 | 54.5 | 0.273 | 80.0 | 77.8 | 81.8 | 0.025 | 80.0 | 77.8 | 81.8 | 0.025 |
| SVM | 55.0 | 55.6 | 54.5 | 0.619 | 85.0 | 88.9 | 81.8 | 0.008 | 85.0 | 88.9 | 81.8 | 0.008 |
| LDA | 65.0 | 22.2 | 100.0 | 0.273 | 65.0 | 44.4 | 81.8 | 0.273 | 70.0 | 44.4 | 90.9 | 0.145 |

LDA – linear discriminant analysis, LR – logistic regression, p – p-value calculated using one-sample binominal test, SVM – linear support vector machines

**Supplementary Material 3: The final statistical model**

The final statistical model based on a single LR classifier enables prediction of response to VNS using the following formula:

$$\frac{1}{1+e^{-\left( -159.09-0.68*g_{1}+2.07*g_{2}-1.22*g_{3}+6.95*g_{4}+0.87*g_{5}-3.61*g_{6}-7.22*g_{7}+3.11*g_{8} \right)}},$$

where g1 is theta_Int3_Fz-F4-Fp2 (i.e., mean EEG value in theta frequency band for the 3^rd^ time interval from electrodes Fz, F4, and Fp2), g2 is theta_Int8_F8-T4 (i.e., mean EEG value in theta frequency band for the 8^th^ time interval from electrodes F8 and T4), g3 is alpha_Int2_O2-P4-T6-Pz, g4 is alpha_Int5_O1-P3-T5-Pz, g5 is beta_Int2_Fz-F4-Fp2, g6 is beta_Int3_Fz-F4-Fp2, g7 is beta_Int5_O1-P3-T5-Pz, and g8 is gamma_Int5_C3-C4-Cz.

The result of the formula is a probability of response. If a patient has probability higher than 50%, he is classified as a Responder, otherwise as a Non-responder. For example, for a patient with $g_{1}$=100.6, $g_{2}$=126.1, $g_{3}$=70.3, $g_{4}$=73.3, $g_{5}$=200.7, $g_{6}$=101.2, $g_{7}$=76.0, $g_{8}$=91.6, the probability will be:

$$\frac{1}{1+e^{-\left( -159.09-0.68*100.6+2.07*126.1-1.22*70.3+6.95*73.3+0.87*200.7-3.61*101.2-7.22*76.0+3.11*91.6 \right)}}=0.864.$$

It means that the patient has 86.4% probability of VNS response and thus will be classified to the group of Responders.

In case of prediction using classifier voting, 60 LR models were created (regression coefficients not shown). Each model labeled a patient as a Responder or a Non-responder. Then the 60 models voted, and the patient was classified as a Responder or a Non-responder based on a majority vote. Besides, mean votes were calculated and used in receiver operating characteristic (ROC) analysis to show sensitivity and specificity for different cut-off levels (Supplementary Figure 3.1). For the used cut-off value of 0.5, sensitivity and specificity was 83.3% and 90.0%, respectively, which corresponds to the results shown in Table 2. The area under the curve (AUC) was equal to 0.858.

**Supplementary Figure 3.1:** **Results of ROC analysis for the validation of the final model**

The plot on the left depicts the ROC curve for the classification of 22 patients from the validation Cohort 2. The table on the right shows estimates of sensitivity and specificity in different cut-off levels. The used cut-off value of 0.5 is enhanced in red.


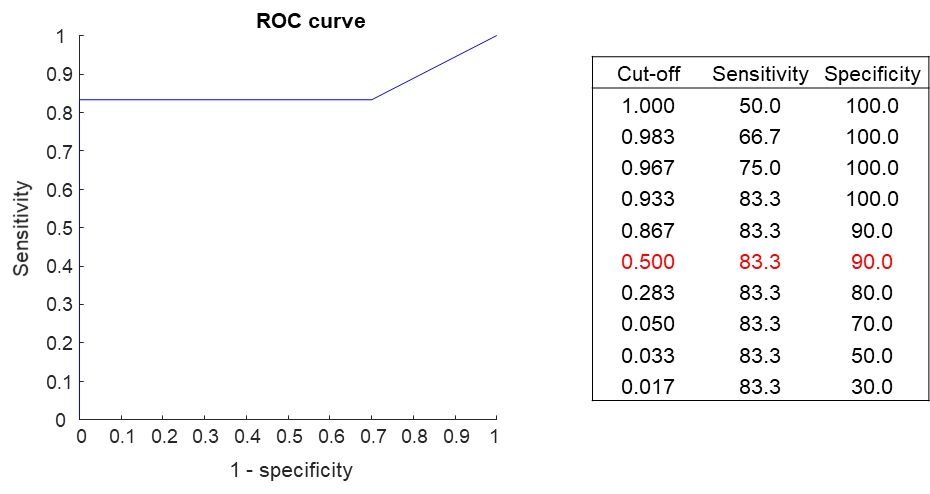

Supplement: Supplementary file 1 [file Data_Sheet_1.docx]
